# Genomic characterization of a *bla*_KPC-2_–producing IncM2 plasmid harboring transposon ΔTn*6296* in *Klebsiella michiganensis*

**DOI:** 10.3389/fcimb.2024.1492700

**Published:** 2024-11-12

**Authors:** Jian-Mei Song, Hu-Bo Long, Mei Ye, Bao-Rui Yang, Guang-Juan Wu, Hong-Chun He, Jun-Ling Wang, Hong-Wei Li, Xiao-Gang Li, De-Yao Deng, Bo Li, Wen-Li Yuan

**Affiliations:** ^1^ Department of Clinical Laboratory, The Affiliated Hospital of Yunnan University (The Second People’s Hospital of Yunnan Province), Kunming, Yunnan, China; ^2^ Department of Clinical Laboratory, Affiliated Qujing Hospital of Kunming Medical University (The First People’s Hospital of Qujing), Qujing, Yunnan, China; ^3^ Department of General Surgery, The Affiliated Hospital of Yunnan University (The Second People’s Hospital of Yunnan Province), Kunming, Yunnan, China

**Keywords:** *Klebsiella michiganensis*, carbapenem-resistant *Enterobacteriaceae*, whole genome sequencing, *bla*_KPC-2_ carbapenemases, IS*Kpn27*-*bla*_KPC-2_-IS*Kpn6*, phylogenetics

## Abstract

*Klebsiella michiganensis* is an emerging hospital-acquired bacterial pathogen, particularly strains harboring plasmid-mediated carbapenemase genes. Here, we recovered and characterized a multidrug-resistant strain, *bla*_KPC-2_–producing *Klebsiella michiganensis* LS81, which was isolated from the abdominal drainage fluid of a clinical patient in China, and further characterized the co-harboring plasmid. *K. michiganensis* LS81 tested positive for the *bla*_KPC-2_ genes by PCR sequencing, with *bla*_KPC-2_ located on a plasmid as confirmed by S1 nuclease pulsed-field gel electrophoresis combined with Southern blotting. In the transconjugants, the *bla*_KPC-2_ genes were successfully transferred to the recipient strain *E. coli* EC600. Whole-genome sequencing and bioinformatics analysis confirmed that this strain belongs to sequence type 196 (ST196), with a complete genome comprising a 5,926,662bp circular chromosome and an 81,451bp IncM2 plasmid encoding *bla*_KPC-2_ (designated pLS81-KPC). The IncM2 plasmid carried multiple β-lactamase genes such as *bla*_TEM-1B_, *bla*_CTX-M-3_, and *bla*_KPC-2_ inserted in truncated Tn*6296* with the distinctive core structure IS*Kpn27*–*bla*_KPC-2_–IS*Kpn6*. A comparison with 46 *K. michiganensis* genomes available in the NCBI database revealed that the closest phylogenetic relative of *K. michiganensis* LS81 is a clinical isolate from a wound swab in the United Kingdom. Ultimately, the pan-genomic analysis unveiled a substantial accessory genome within the strain, alongside significant genomic plasticity within the *K. michiganensis* species, emphasizing the necessity for continuous surveillance of this pathogen in clinical environments.

## Introduction

Carbapenem-resistant *Enterobacteriaceae* (CRE) infections are characterized by increased prevalence, severe disease manifestations, longer hospital stays, higher healthcare costs, and increased mortality rates compared to susceptible carbapenem infections, thereby exacerbating a considerable global burden (Perez and Bonomo, 2019). In the 2024 updated WHO Bacterial Priority Pathogens List, CRE is classified as a critical group due to their ability to transfer resistance genes, which have been an urgent and continuous threat to global public health. An important mechanism for carbapenem resistance in CRE is the production of carbapenemases such as *K. pneumoniae* carbapenemase (KPC). KPC is a class A serine β-lactamase that belongs to group 2f in the Bush classification. KPC-2 is the most commonly identified variant that can efficiently hydrolyze all β-lactam antimicrobials, including carbapenems (Ding et al., 2023). The drastic increase in antibiotic-resistant strains exacerbates the failure to cure, posing a significant challenge to clinical anti-infective treatments.

*Klebsiella michiganensis* is an emerging species of pathogenic *Klebsiella*, one of the nine species of *Klebsiella oxytoca* complex (Yang et al., 2022). *K. michiganensis* has continued to spread in clinical settings, colonizing patients and causing nosocomial infections since it was first discovered in a toothbrush holder in 2012 (Saha et al., 2013). The clonal background of clinical isolates of *K. michiganensis* remains poorly understood and accurate species identification relies on genome sequencing and analysis. The chromosome-encoded OXY-type β-lactamase in *K. michiganensis* confers resistance to amino- and carboxy-penicillins. This species also acquires extended-spectrum β-lactamases (ESBLs) and carbapenemases through horizontal gene transfer (HGT), similar to other *Enterobacteriaceae* (Campos-Madueno et al., 2021). They also cause infections with severe clinical outcomes in hospitalized patients and can even lead to the outbreaks of clonal isolates (Chapman et al., 2020).

Carbapenemase genes can be horizontally transferred between resistant and susceptible strains via transposons and conjugative plasmids, enabling the rapid spread of resistance genes among *Enterobacteriaceae* and contributing to increasing CRE infections. Tn*4401* is a removable element that commonly carries the *bla*_KPC-2_ gene in Europe, Brazil, and the United States (Furlan et al., 2023). In Asia, *bla*_KPC-2_ is mainly located on different variants of Tn*1721*-like transposons (Tang et al., 2017). Typical transposons Tn*4401* and Tn*1721*, both members of the Tn*3* family, have been demonstrated to mobilize the *bla*_KPC-2_ at high transposition frequencies (Tang et al., 2022). Here, we characterized a *bla*_KPC-2_–producing IncM2 plasmid harboring transposon ΔTn*6296* in *K. michiganensis* LS81 recovered from a patient at a university-affiliated hospital in China. The ability of this strain to transfer the *bla*_KPC-2_ genes was assessed by conjugation experiments. The genetic structure and location of the resistance genes on the IncM2 plasmid were analyzed through S1 nuclease pulsed-field gel electrophoresis (S1-PFGE) and whole-genome sequencing (WGS). Our comparison with the whole-genome sequence available from NCBI of *K. michiganensis* strains carrying *bla*_KPC-2_ aimed to discern the driving force behind the *bla*_KPC-2_ IncM2 plasmids.

## Materials and methods

### Patient characteristics and strain screening

A 65-year-old Chinese man admitted to the Affiliated Hospital of Yunnan University (Kunming, China) with no history of travel outside China was diagnosed with severe pneumonia in 2021, and strain LS81 was isolated in the clinical laboratory from this patient’s abdominal drainage fluid in February. *Klebsiella oxytoca* was initially identified as the pathogen from his culture by matrix-assisted laser desorption/ionization time-of-flight mass spectrometry (MALDI-TOF/MS) (Bruker Daltonik GmbH, Bremen, Germany).

### Antimicrobial susceptibility testing and carbapenemase-producing organism characterization

Antimicrobial susceptibility testing of cefoperazone-sulbactam, piperacillin-tazobactam, amoxicillin-clavulanic acid, ceftazidime,ceftriaxone, cefepime, ertapenem, imipenem, amikacin, levofloxacin, trimethoprim-sulfamethoxazole, and cefuroxime was done using the bioMérieux VITEK2. Minimum inhibitory concentration (MIC) values were interpreted according to Clinical and Laboratory Standards Institute document M100-S32 (Clinical and Laboratory Standards Institute, 2022). Quality control was conducted using the *E. coli* ATCC 25922 and *K. pneumoniae* ATCC BAA-1705. The modified carbapenem inactivation method (mCIM) in conjunction with the EDTA-modified carbapenem inactivation method (eCIM) test was utilized for phenotypic detection of carbapenemase enzymes. PCR amplification was employed to identify major carbapenemase resistance genes (*bla*_KPC-2_, *bla*_NDM-1_, *bla*_OXA-48_, *bla*_VIM-2_, and *bla*_IMP_).

### Conjugl transfer and location of *bla*_KPC-2_ gene

The transferability of the *bla*_KPC-2_ gene was determined by the broth mating conjugal method, using the previously described protocol with some adjustments (Prah et al., 2021). The LS81 strain was mixed with rifampicin-resistant *E. coli* EC600 in a volume ratio of 1:2, with LS81 as donor strain and *E. coli* EC600 as recipient strain. The plates were incubated at 35°C overnight, and transconjugants were selected on Mueller-Hinton agar plates containing rifampicin (600 μg/mL) and imipenem (1 μg/mL; Solarbio, Beijing, China). PCR sequencing confirmed the presence of the plasmid carrying *bla*_KPC-2_. To determine the location of the *bla*_KPC-2_ gene in the LS81 strain, we performed pulsed-field gel electrophoresis (PFGE) after S1 restriction enzyme digestion. This was followed by Southern blotting with a digoxigenin-labeled KPC-2 gene probe to localize and fingerprint the plasmid, based on a previous report with some modifications (Bi et al., 2018). DNA isolation was conducted on 1% agarose gel (SeaKem Gold) in 0.5× Tris-borate-EDTA buffer (TBE) in the CHEF-Mapper XA system (Bio-Rad, Hercules, CA, USA) for 18 h, under the following conditions: field strength of 6 V/cm², angles of 120°, initial switching time of 2.2 s, final pulse time of 63.8 s, and temperature of 14°C.

### Whole-genome sequencing

Genomic DNA was extracted by SDS extraction combined with column purification. The LS81 strain’s whole genome was sequenced by both Illumina Novaseq 6000 (Illumina, San Diego, CA, United States) and Oxford Nanopore PromethION 24 (Nanopore, Oxford, United Kingdom) sequencing technology platforms. Unicycler (Wick et al., 2017) assembled high-accuracy Illumina reads (Q30 > 85%) to obtain high-quality bacterial genome backbones and Nanopore data connected high-quality contig, and Pilon (Walker et al., 2014) was used to correct assembled genomes.

### Genome component prediction, sequence annotation, and protein classification

RAST 2.0 (Overbeek et al., 2014) combined with BLAST database against UniProtKB/Swiss-Prot (The UniProt Consortium, 2023) and RefSeq (O’leary et al., 2016) databases was used to predict open reading frames and pseudogenes. Annotation of resistance genes, multilocus sequence typing (MLST), and plasmid replicon types was done using bioinformatics tools from the Center for Genomic Epidemiology (Zankari et al., 2012). Plasmids were analyzed for removable element information using ISfinder (Siguier et al., 2006) and Tn Number Registry (Tansirichaiya et al., 2019). Finally, the data were visualized using software R version 4.0.4 and Scalable Vector Graphics (SVG) 1.1 to further map the genetic background.

### Phylogenetic analysis

To explore the global genomic epidemiology of *K. michiganensis* strains carrying *bla*_KPC-2_, we conducted phylogenetic analysis between strain LS81 and other *K. michiganensis* strains carrying *bla*_KPC-2_, using data from the NCBI GenBank database to 6 May 2024. We obtained genome sequences and strain metadata for 993 isolates of *K. michiganensis*, of which 49 strains were found to carry *bla*_KPC-2_ resistance gene. After excluding three potentially contaminated strains, we conducted a phylogenetic analysis on the remaining 46 strains (**Supplementary Table S1**). *Klebsiella pneumoniae* HS11286 was used as an outgroup in the analysis because of its close phylogenetic group. OrthoFinder (Emms and Kelly, 2015) clustered protein sequences into orthologous protein families, screened 2,924 single-copy gene families for phylogenetic analysis, and used MAFFT (Katoh et al., 2019) to align protein sequences, iteratively comparing 1,000 replicates. TrimAI (Capella-Gutiérrez et al., 2009) trimmed sequences and SeqKit (Shen et al., 2024) integrated with these trimmed alignment results of 2,924 protein families into a super-sequence matrix, finally, Iqtree2 (Minh et al., 2020) constructed a maximum likelihood tree.

### Data availability

All reads generated in this study were deposited in the NCBI GenBank database. The genome sequences of the *K. michiganensis* LS81 chromosome and plasmid pLS81-KPC were submitted to GenBank with the accession numbers CP089448 and CP089449. The phylogenetic analysis used *Klebsiella pneumoniae* HS11286 as the outgroup genome, with the NCBI RefSeq assembly number GCF_000240185.1.

## Results

### Bacterial identification, antimicrobial susceptibility testing, and conjugal transfer of *bla*_KPC-2_

Strain LS81 was found to be mCIM-positive and eCIM-negative. Subsequently, the *bla*_KPC-2_ gene was identified in this strain through sequencing of the PCR-positive products. It was initially misidentified as *K. oxytoca* by MALDI-TOF/MS and finally reclassified as *K. michiganensis* by WGS. In accordance with the CLSI breakpoint criteria, strain LS81 was resistant to almost all β-lactam antibiotics tested, including carbapenems (imipenem), cefuroxime, ceftriaxone, ceftazidime, cefepime, cefoperazone-sulbactam, piperacillin-tazobactam, and amoxicillin-clavulanic acid, whereas it was susceptible to quinolones (amikacin and levofloxacin) and trimethoprim-sulfamethoxazole (**Table 1**). Conjugl transfer showed that strain LS81 successfully transferred *bla*_KPC-2_ into *E. coli* EC600, as confirmed by PCR sequencing. The transconjugate was the *bla*_KPC-2_–encoding *E. coli* EC600 (designated H-LS81), which had similar antibiotic resistance to strain LS81 (**Table 1**).

**Table 1:** Minimum inhibitory concentration(MIC) of antimicrobials for *K. michiganensis* LS81 and transconjugant H-LS81.

	MIC (μg/mL)											
	CPS	TZP	AMC	CAZ	CRO	FEP	ETP	IPM	AMK	LEV	SXT	CXM
LS81	**≥64**	**≥128**	**≥32**	**≥16**	**≥64**	**≥16**	1	**≥16**	≤2	≤0.12	≤20	**≥64**
H-LS81	**≥64**	**≥128**	**≥32**	**32**	**≥64**	**≥16**	1	**8**	≤2	0.5	≤20	**≥64**

Non-susceptible values are marked in boldface. CPS, cefoperazone-sulbactam; TZP, piperacillin-tazobactam; AMC, amoxicillin-clavulanic acid; CAZ, ceftazidime; CRO, ceftriaxone; FEP, cefepime; ETP, ertapenem; IPM, imipenem; AMK, amikacin; LEV, levofloxacin; SXT, trimethoprim-sulfamethoxazole; CXM, cefuroxime.

### Genomics features of *K. michiganensis* LS81

Accurate species identification depends on genome sequencing and analysis. The genome of *K. michiganensis* LS81 consists of a circular chromosome of 592662 bp and a plasmid. The average G+C content of the chromosomes was 56.12%, which contained 5,458 protein-coding genes, 25 ribosomal RNAs (rRNAs), and 84 transfer RNAs (tRNAs). Screening for drug resistance gene information revealed that the chromosome contains the *bla*_OXY-1-3_ gene and that the plasmid pLS81-KPC carries the β-lactam antibiotic resistance genes (ARGs): *bla*_KPC-2_, *bla*_TEM-1B_, and *bla*_CTX-M-3_ (**Table 2**).

**Table 2:** Genomics features of *K. michiganensis* LS81.

Feature	Chromosome	pLS81-KPC
Total number of bases (bp)	5,926,662	81,451
G+C content (%)	56.12%	52.36%
No. of protein-coding sequences	5,458	111
No. of rRNA genes	25	0
No. of tRNA genes	84	0
No. of ncRNA genes	9	0
plasmid replicon type	–	IncM2
Resistance genes	*bla*_OXY-1-3_	*bla*_KPC-2_, *bla*_TEM-1B_, and *bla*_CTX-M-3_
Accession numbers	CP089448.1	CP089449.1

### Characterization of plasmid pLS81-KPC

**Figure 1:**
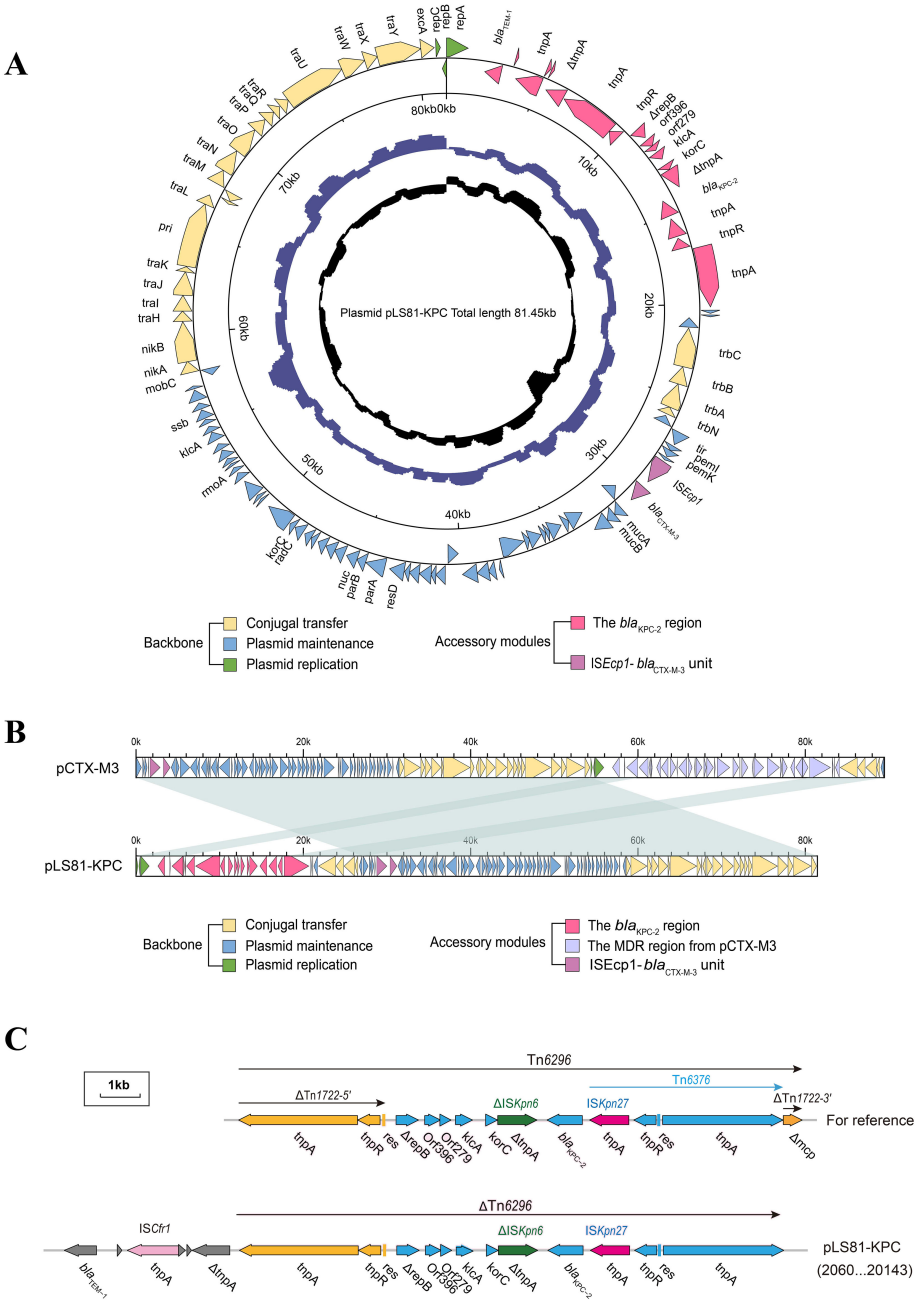
Genes are indicated by arrows; backbone and accessory modules are colored differently. Shading denotes regions of homology (>95% nucleotide identity). **(A)** Circular map of pLS81-KPC. The intermediate ring and the innermost ring represent the GC content and the GC-skew [(G − C)/(G + C)]. **(B)** Linear comparison of the IncM2 plasmids pLS81-KPC and pCTX-M3. **(C)** Linear comparison of the Tn*6296* and the genetic structure surrounding the *bla*_KPC-2_ region in the plasmids pLS81-KPC.

S1-PFGE and Southern blotting showed the *bla*_KPC-2_ gene located on a plasmid of approximately ~80-kb (**Supplementary Figure S1**). WGS confirmed that the pLS81-KPC plasmid was a circular DNA of 81,451-bp with 111 open reading frames and an average G+C content of 52.36%. The replication initiation region is composed of the repABC family, and the stable region is mainly composed of an independent part of the long 27.2-kb segment, including pemI, pemK, mucA, mucB, parA, and parB. The conjugation transfer region consists of two distant tra regions (22.8-kb) and a trb region (4.7-kb) of different lengths (**Figure 1A**). Incompatibility plasmid classification showed that pLS81-KPC is an IncM2-type plasmid, having the same replication subtype as the reference plasmid pCTX-M3 (accession number: AF550415), two plasmids with 77% coverage, 99% nucleotide consistency, and the stable region and the conjugation transfer region were essentially the same (**Figure 1B**). The exogenous insertion region of pLS81-KPC was mainly composed of two antibiotic-resistant regions: the *bla*_KPC-2_ region and IS*Ecp1*-*bla*_CTX-M-3_ unit. Located between the repABC replication initiation region and the trb plasmid conjugation transfer region, the full length of the *bla*_KPC-2_ region is about 16.7-kb. In the IS*Ecp1*-*bla*_CTX-M-3_ transposable unit, because there is no obvious site selection for IS*Ecp1* insertion, they move as an integral mobile element, facilitating *bla*_CTX-M_ spread and replication. The *bla*_KPC-2_ gene lies between IS*Kpn27* and IS*Kpn6*, forming an IS*Kpn27*–*bla*_KPC-2_–IS*Kpn6* core structure. As Tn*1722-3′* is truncated, it forms a variant of Tn*6296*; the ΔTn*6296* transposon constituted the core genetic environment of *bla*_KPC-2_ (**Figure 1C**).

### Phylogenetic trees of carrying *bla*_KPC-2_ *K. michiganensis*

**Figure 2:**
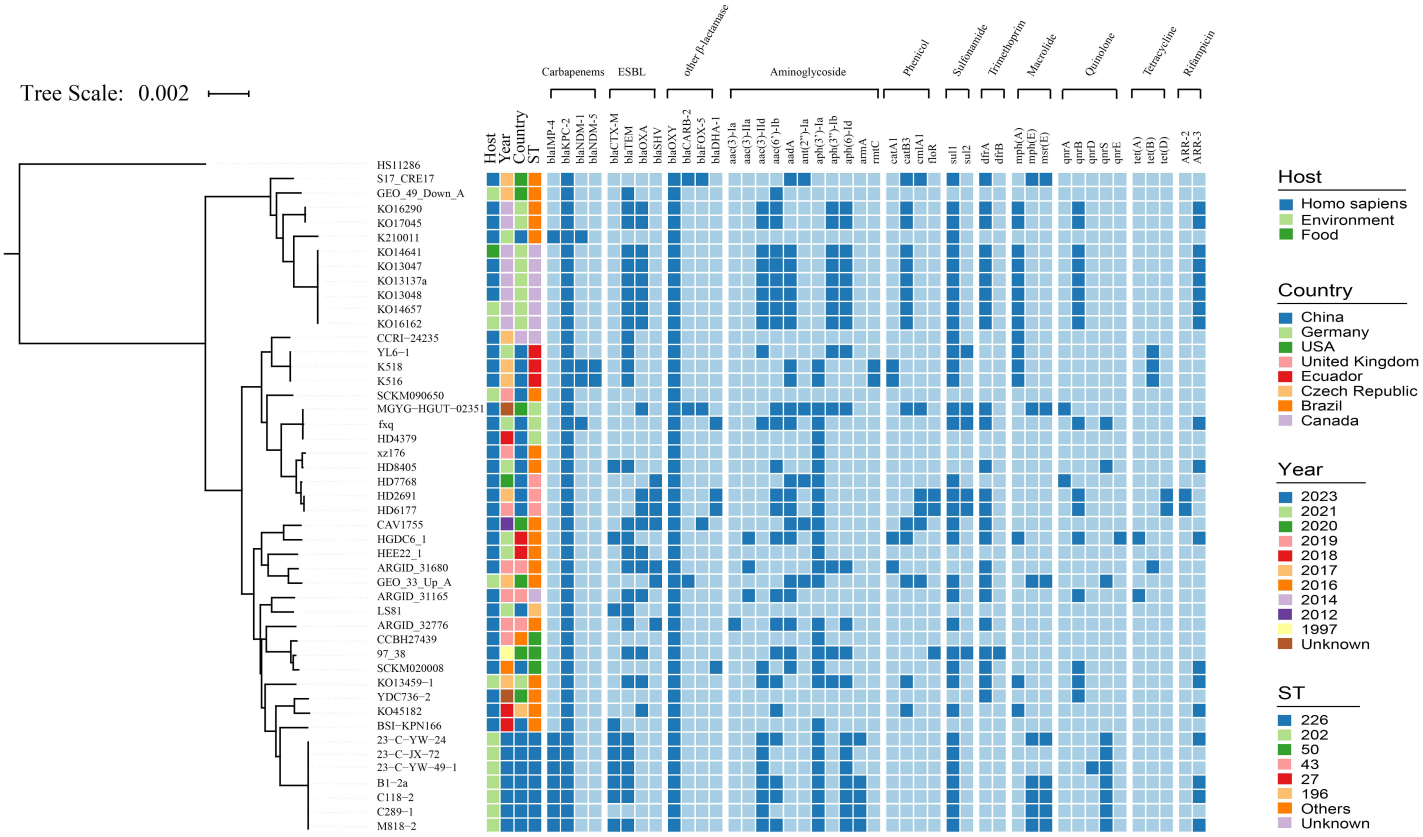
Maximum-likelihood phylogenetic tree of KPC-2-producing *Klebsiella michiganensis* LS81 and 45 other *K. michiganensis* isolates based on core genomes. *Klebsiella pneumoniae* HS11286 used as outgroup in analysis. The figure provides information on the antimicrobial resistance genes, host, country, date of collection, and MLST type of these strains. Detailed information on the isolates included in this study is summarized in **Supplementary Table S1**.

To correlate the genetic information of *K. michiganensis* strains carrying *bla*_KPC-2_, phylogenetic tree analysis based on protein sequences was performed to further explore their population structure, evolutionary relationships, and internal genome structure. We collected 46 *K. michiganensis* genomes that had average nucleotide identity (ANI) values of ≥95% based on *K. michiganensis* reference strain THO-011. A total of 46 *K. michiganensis* hosts from 32 Homo sapiens, 13 environments, and one food are shown in **Figure 2** and **Supplementary Table S1**. They came from different countries, mainly from China (n = 22), followed by Germany (n = 9), the USA (n = 7), the United Kingdom (n = 3), and Ecuador (n = 2), the remaining three strains were from Brazil, the Czech Republic, and Canada, and dated from 1997 to 2023. Phylogenetic analysis showed that *K. michiganensis* carrying *bla*_KPC-2_ could be divided into two major clades on the tree, with the second clade further subdivided into two subclades. It is noteworthy that *K. michiganensis* LS81 and ARGID_31165 share a common ancestral node, show an unequivocal bootstrap value of 100% through the neighbor-joining analysis, and indicate that *K. michiganensis* LS81 may have evolved from a subspecies of this strain, ARGID_31165 from a homo sapiens wound swab from the United Kingdom 2019. Further analysis of the genomic information showed that, except for *bla*_KPC-2_, *K. michiganensis* LS81 and ARGID_31165 contained *bla*_OXY_ and *bla*_TEM-1_ genes, but, as different variants, they had no common plasmid replicon type and MLST type.

We detected 152 plasmids with 35 subtypes of inc gene variants, grouped into 11 different types: IncF, IncH, IncN, IncM, IncU, IncX, IncP, IncC, IncR, IncQ, and Col; the most frequently detected inc types were IncFII, IncFIB, and IncN (**Supplementary Table S1**). We investigated the drug resistance profiles of *K. michiganensis* strains and identified a total of 44 resistance genes (**Figure 2**). Except for the *bla*_KPC-2_ gene, *bla*_OXY_ is prevalent in all strains (46/46) and exists as *bla*_OXY_ variants (*bla*_OXY-1_, *bla*_OXY-2_, and *bla*_OXY-5_), followed by sul1 in sulfonamide (35/46). *bla*_CTX-M_, *bla*_TEM_, *bla*_OXA_, and *bla*_SHV_ were detected in 36 strains as major ARGs for ESBLs. We screened for the gene encoding carbapenem resistance, and 11 strains even carried other carbapenemase genes such as *bla*_IMP-4_, *bla*_NDM-1_, and *bla*_NDM-5_.

## Discussion

The increasing prevalence and spread of the carbapenem resistance gene *bla*_KPC-2_ poses a serious threat to public health, the *bla*_KPC-2_ gene has been widely detected in different hosts. In recent years, other *Enterobacteriaceae* strains producing KPC-2 have been identified, including *Enterobacter cloacae*, *Citrobacter freundii*, *Serrella marcescens*, and *Protesus mirabilis* (Stewardson et al., 2019). *K. michiganensis* can colonize in the skin, mouth, intestines, and respiratory tract of healthy individuals and patients, and it is considered an opportunistic pathogen causing clinical infections. The extant studies highlight its critical clinical importance as a nosocomial pathogen, particularly in patients with underlying conditions causing multiple infections (Meng et al., 2022). Research has reported a propensity for *K. michiganensis* to cause bloodstream infections in immunocompromised individuals and severe, potentially fatal gastrointestinal damage in premature infants (Chen et al., 2020).

Nonetheless, research into the full pathological implications of *K. michiganensis* remains limited. Owing to the capacity of MALDI-TOF/MS to rapidly identify the species level of pathogenic microorganisms, its application in clinical microbiology laboratories has proliferated in recent years. However, the reference spectra for these novel bacterial species may be absent from most laboratories’ MALDI-TOF/MS databases, complicating the differentiation of each species within the *K. oxytoca* complex. In some cases, distinguishing *K. michiganensis* from the *K. oxytoca* complex requires reliance on genomic analysis techniques such as WGS, which are not readily available in standard clinical laboratories, posing a significant challenge to accurate *Klebsiella* species identification. As *K. michiganensis* is often mistakenly identified as *K. oxytoca*, it is conceivable that some clinical conditions ostensibly due to *K. oxytoca* may actually be caused by *K. michiganensis*.

The *bla*_KPC-2_ gene can be transferred by plasmids, transposons, integrons and so on, and the critical step is HGT of mobile genetic elements (MGEs) around this determinant. Plasmids are MGEs that can replicate independently of the chromosome, and many carry genes encoding beneficial phenotypes for the survival of host strains, such as antimicrobial resistance (AMR) and virulence (Grodner et al., 2024). The IncM2 plasmid carrying *bla*_KPC-2_ in *K. michiganensis* LS81 could be autonomously transferred into *E. coli* EC600, the resistance phenotype of the conjugate was obtained by the pLS81-KPC plasmid. It is noteworthy that *K. michiganensis* LS81 and the transconjugant H-LS81 have similar drug resistance, and the transconjugant strain once cloned will pose significant clinical challenges.

Conjugative plasmids are considered to be the primary vectors for the dissemination of AMR because of their ability to transfer horizontally between bacteria, ensure their stability through toxin-antitoxin and distribution systems, and offset their own fitness costs through mutation (Partridge et al., 2018). Among the 46 *bla*_KPC-2_–producing *K. michiganensis* genomes included in our study, 35 different plasmid subtypes were detected, with the most common Inc types being IncFII, IncFIB, and IncN. Similar to the report on the *K. oxytoca* complex, *bla*_KPC-2_ is mainly found on IncN or IncF plasmids (Yang et al., 2022). The diversity of plasmid backgrounds in these isolates may provide opportunities for *bla*_KPC-2_ exchange between different plasmid families. IncL/M plasmids were previously classified in the same incompatibility group, but, after verification that they were compatible with each other, IncL and IncM plasmids were separated into two different incompatibility groups with an overall nucleotide identity of about 94% (Getino et al., 2022). IncM plasmid poses a threat due to its broad host range and increasing prevalence in clinical, animal, and environmental isolate (Rozwandowicz et al., 2018). In our study, *K. michiganensis* pLS81-KPC carrying *bla*_CTX-M3_ had almost the same stable region and conjugal transfer region as the IncM2 reference plasmid pCTX-M3, derived from a *Citrobacter freundii* isolate from Poland (Gołebiewski et al., 2007). As reported, the IncM pCTX-M-3 plasmid can even combine with other plasmid systems to exhibit more complex replication and transfer behavior, while the range of recipients of its conjugation system is broad, including α-, β-, and γ-proteobacteria and also Firmicutes (Kern-Zdanowicz, 2021).

In investigating neonatal sepsis in several low- and middle-income countries within Africa and South Asia, it has been found that diverse strains shared identical plasmid types and exhibited the same carbapenemase gene variants, indicating the potential occurrence of successful dissemination and acquisition across multiple species (Sands et al., 2021). Moreover, the discovery of identical carbapenemase ARGs across diverse plasmids substantiates the extensive dissemination of AMR (Brandt et al., 2019). In Germany, researchers observed a widespread distribution of the *bla*_KPC-2_ gene across various species, a process enabled by both intra- and inter-species HGT involving a unique IncN plasmid; this transfer also resulted in the rise of highly antibiotic-resistant bacteria that possess up to 14 additional ARGs encoded on the same plasmid (Yao et al., 2024). These findings underscore the pivotal role of MGEs in enabling the worldwide dissemination of resistance via HGT among a diverse array of bacterial species.

In genetic variation and evolution of bacteria, transposable elements play an important role. The *bla*_KPC-2_ gene is mainly located in the Tn*4401* and Tn*1721* transposable elements. Deletions or insertions at different sites in Tn*4401* divide the gene into subtypes; there are at least nine subtypes of Tn*4401a*–Tn*4401I*, of which Tn*4401a* and Tn*4401b* are the most common (Araújo et al., 2018). The Tn*1721* variant has been reported to be the dominant structure in Asia, particularly in China, and the structure of the Tn*1721* variant in the *bla*_KPC-2_ epidemic is complex and diverse. In 2016, a scientific research team in China studied the genetic environment of the *bla*_KPC-2_ gene in *Klebsiella pneumoniae* isolates and found that *bla*_KPC-2_ is located in two types of genetic structures, Tn*1721*–*bla*_KPC-2_–Tn*3* and Tn*1721*– *bla*_KPC-2_–ΔTn*3*-IS*26* (Shen et al., 2016). In 2022, this team again reported that transposition of the Tn*1721* variant promoted the evolution and spread of the KPC-2 plasmid in *Enterobacteriaceae*, *bla*_KPC-2_ can be located on three different variants of Tn*1721*, A1-type (Tn*1721*-*bla*_KPC-2_), A2-type (Tn*1721*–*bla*_KPC-2_–IS*26*), and B-type (Tn*1721*–*bla*_KPC-2_–IRL2) (Tang et al., 2022).

Tn*6296*, a transposon of the Tn*3* family originally identified in the *Klebsiella pneumoniae* plasmid pKP048 (Jiang et al., 2010). The core structure of Tn*6376*–*bla*_KPC-2_–ΔIS*Kpn6*-korC-klcA-orf279-orf396-ΔrepB was inserted into Tn*1722*, truncated and split into ΔTn*1722-5′* and ΔTn*1722-3′*. Unlike the most common *bla*_KPC-2_–producing mobile element transposon, the Tn*6296*/Tn*6376* chimera may have evolved by recombination of the Tn*4401*. Insertions and deletions in the exogenous insertion region *bla*_KPC-2_ lead to different ΔTn*6296* variant structures, and different lengths of truncated Tn*6296* can be observed on different plasmids. Tn*6296*-derived complex transposons are present in common species of *Enterobacteriaceae*, such as *Klebsiella pneumoniae* pHS062105-3 (Shanghai, China, KF623109), *Klebsiella pneumoniae* pVA833-92 (Santiago de Chile, Chile, CP093457), and many other bacterial species such as *Serratia marcescens* pKPC-2-HENAN1602 (Zhengzhou, China, CP047392) (Wang et al., 2022b), *Citrobacter freundii* PP10159-3 (Chongqing, China, MF072963) (Ouyang et al., 2018), *Aeromonas hydrophila* PK522-A-KPC and pK522-B-KPC (Taizhou, China, CP118700, CP118703) (Jing et al., 2024), *Citrobacter koseri* pCK1008-KPC-123 (Hangzhou, China, ON209376) (Wang et al., 2022a), *Aeromonas media* pE31A (Jinan, China, CP067418), and *Klebsiella aerogenes* pC212158-KPC_75k (Hangzhou, China, CP139398) (Li et al., 2024). Tn*6296*-derived complex transposons show diversity in bacterial clones carrying the *bla*_KPC-2_ gene. Notably, they share a common core structure IS*Kpn27*–*bla*_KPC-2_–IS*Kpn6*, we speculate that IS*Kpn27*–*bla*_KPC-2_–IS*Kpn6* may be the main genetic structure in the gene microenvironment carrying *bla*_KPC-2_. These transposons not only carry the *bla*_KPC-2_ gene but can also contain other ARGs, forming complex chimeric structures for rapid adaptation and evolution under antibiotic selection pressure.

## Conclusion

In conclusion, this study is the first to characterized a *bla*_KPC-2_–producing IncM2 plasmid pLS81-KPC harboring transposon ΔTn*6296* in *K. michiganensis* strain in China, suggesting that it may play an important role in the dissemination of *bla*_KPC-2_ in *K. michiganensis*. In addition, the presence and significance of *K. michiganensis* in clinical pathology may be underestimated because most strains designated as *K. oxytoca* in research have not been subjected to accurate species identification. Consequently, this species might be implicated in diseases previously attributed to the *K. oxytoca* complex and other members of the genus, with the epidemiology of colonization and infection of each species in humans remaining largely uncharted. Although China is considered an endemic area for CRE, large-scale molecular epidemiological studies of the carbapenem-resistant *K. oxytoca* complex have been rare in China. As an emerging pathogen, *K. michiganensis* has relatively few reported clinical infections and its genomic characteristics and multidrug resistance profile need further investigation.

## Data Availability

The datasets presented in this study can be found in online repositories. The raw data from the sequence experiment have been deposited in the database of NCBI under accession numbers CP089448 and CP089449. (https://www.ncbi.nlm.nih.gov/).
