# Supplementary material for: Genomic characterization of a bla KPC-2–producing IncM2 plasmid harboring transposon ΔTn6296 in Klebsiella michiganensis
Source: Front Cell Infect Microbiol. 2024 Nov 12;14:1492700. doi: 10.3389/fcimb.2024.1492700 (PMC11588702; doi:10.3389/fcimb.2024.1492700)
Supplement: Supplementary file 2 [file Table2.docx]

**Supplementary Figure S1: S1-PFGE and Southern blot hybridization**


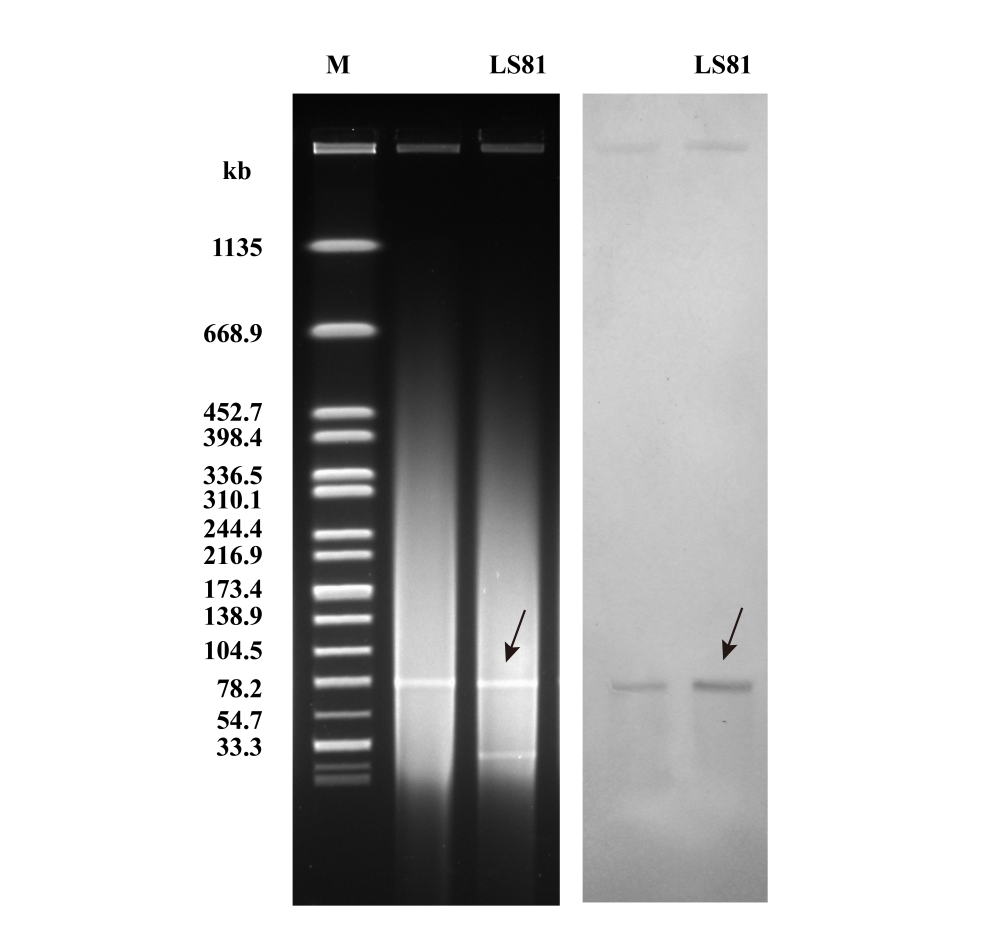


Marker: *Salmonella enterica*, serotype Braenderup H9812 digested by XbaI restriction enzyme. The black arrows are the positive signals via southern blot hybridization of *Klebsiella michiganensis* LS81 with *bla*_KPC-2_ specific probe.
